# Guidewire exchange vs new site placement for temporary dialysis catheter insertion in ICU patients: is there a greater risk of colonization or dysfunction?

**DOI:** 10.1186/s13054-016-1402-6

**Published:** 2016-07-30

**Authors:** Elisabeth Coupez, Jean-François Timsit, Stéphane Ruckly, Carole Schwebel, Didier Gruson, Emmanuel Canet, Kada Klouche, Laurent Argaud, Julien Bohe, Maïté Garrouste-Orgeas, Christophe Mariat, François Vincent, Sophie Cayot, Olivier Cointault, Alain Lepape, Michael Darmon, Alexandre Boyer, Elie Azoulay, Lila Bouadma, Alexandre Lautrette, Bertrand Souweine

**Affiliations:** 1 Medical Intensive Care Unit, University Hospital of Clermont-Ferrand, 58 rue Montalembert, 63000 Clermont-Ferrand, France; 2 IAME UMR 1137 Inserm Université Paris Diderot, Paris, F-75018 France; 3 Medical and Infectious Diseases ICU -Paris Diderot University / Bichat Hospital, Paris, France; 4 U823 “Outcome of Cancers and Critical Illness,” Albert Bonniot Institute, La Tronche, France; 5 Medical Intensive Care Unit, Pellegrin Teaching Hospital, University Hospital of Bordeaux, Bordeaux, France; 6 Medical Intensive Care Unit, Saint Louis Teaching Hospital, Paris, France; 7 Medical Intensive Care Unit, Lapeyronie Teaching Hospital, University Hospital of Montpellier, Montpellier, France; 8 Medical Intensive Care Unit, Edouard Herriot Teaching Hospital, University of Lyon, Lyon, France; 9 Medical Intensive Care Unit, University Hospital of Lyon, Lyon, France; 10 Critical Care Medicine Unit, Saint-Joseph Hospital, Paris, France; 11 Nephrology and Critical Care Unit, Nord Teaching Hospital, University of Saint Etienne, Saint Etienne, France; 12 Medical Intensive Care Unit, Avicenne Teaching Hospital, Paris, France; 13 Department of Anaesthesiology and Critical Care Medicine, University Hospital of Clermont-Ferrand, Clermont-Ferrand, France; 14 Nephrology and Critical Care Medicine, Rangueil Teaching Hospital, University of Toulouse, Toulouse, France; 15 Department of Anaesthesiology and Critical Care Medicine Pierre Benite Teaching Hospital, University Hospital of Lyon, Lyon, France; 16 Medical Intensive Care Unit, Nord Teaching Hospital, University of Saint Etienne, Saint Etienne, France; 17 Medical Intensive Care Unit, Bichat-Claude Bernard Teaching Hospital, Paris, France

**Keywords:** Acute kidney injury (AKI), Catheter-related infection, Double lumen vascular catheter, Guidewire exchange versus new venipuncture, Catheter dysfunction, Intensive care unit

## Abstract

**Background:**

Intensive care unit (ICU) patients require dialysis catheters (DCs) for renal replacement therapy (RRT). They carry a high risk of developing end-stage renal disease, and therefore their vascular access must be preserved. Guidewire exchange (GWE) is often used to avoid venipuncture insertion (VPI) at a new site. However, the impact of GWE on infection and dysfunction of DCs in the ICU is unknown. Our aim was to compare the effect of GWE and VPI on DC colonization and dysfunction in ICU patients.

**Methods:**

Using data from the ELVIS randomized controlled trial (RCT) (1496 ICU adults requiring DC for RRT or plasma exchange) we performed a matched-cohort analysis. Cases were DCs inserted by GWE (n = 178). They were matched with DCs inserted by VPI. Matching criteria were participating centre, simplified acute physiology score (SAPS) II +/-10, insertion site (jugular or femoral), side for jugular site, and length of ICU stay before DC placement. We used a marginal Cox model to estimate the effect of DC insertion (GWE vs. VPI) on DC colonization and dysfunction.

**Results:**

DC colonization rate was not different between GWE-DCs and VPI-DCs (10 (5.6 %) for both groups) but DC dysfunction was more frequent with GWE-DCs (67 (37.6 %) vs. 28 (15.7 %); hazard ratio (HR), 3.67 (2.07–6.49); *p* < 0.01). Results were similar if analysis was restricted to DCs changed for dysfunction.

**Conclusions:**

GWE for DCs in ICU patients, compared with VPI did not contribute to DC colonization or infection but was associated with more than twofold increase in DC dysfunction.

**Trial registration:**

This study is registered with ClinicalTrials.gov, number NCT00563342. Registered 2 April 2009.

**Electronic supplementary material:**

The online version of this article (doi:10.1186/s13054-016-1402-6) contains supplementary material, which is available to authorized users.

## Background

Acute kidney injury (AKI) predisposes to end-stage renal disease (ESRD) [1, 2], and the preservation of the vascular network in the event of subsequent chronic dialysis is of foremost importance and may be challenging when short-term dialysis catheter (DC) placement is required for the provision of renal replacement therapy (RRT). DCs are often removed because of suspected infection or dysfunction [3–13].

DC replacement is classically carried out by *de novo* percutaneous venipuncture insertion (VPI) but is not always achievable in cases of obesity, thrombocytopoenia, coagulopathy and extensive burns. In addition, VPI may compromise future vascular access. Guidewire exchange (GWE) is an alternative approach for easily changing DCs and has a lower risk of mechanical complications than VPI at new sites. However, GWE may predispose to infectious complications and is therefore discouraged in central venous catheterization [14].

In patients with chronic haemodialysis who need DC replacement, GWE may be appropriate when other insertion sites are not available or when the risk of a new venipuncture exceeds the benefit of DC removal [15]. This recommendation is for patients with long-term DCs and may not be applicable to critically ill patients on RRT. Of note, Kidney Disease Improving Global Outcome (KDIGO) practice guidelines for AKI provide no information on DC placement by GWE [16]. Of the numerous studies that have recently assessed DC infection in acutely ill patients [3–13, 17] only one, with a small sample size population, looked at the risk of infectious complications following GWE and did not deal with DC dysfunction [12].

We designed a post-hoc cohort study to compare the risk of DC colonization and DC dysfunction after insertion at a new site or GWE. We used data collected prospectively during a randomized controlled trial (Ethanol lock and risk of hemodialysis catheter infection in critically ill patients (ELVIS): ClinicalTrial.gov Registration NCT 00875069) [13].

## Method

### Study patients

The ELVIS trial was a multicentre, randomized, double blind, placebo-controlled, parallel- group study of 1460 critically ill adults from 16 ICUs, who required a temporary DC, which showed that a 2-minute ethanol lock does not decrease the frequency of DC infection [13]. The Sud-Est 1 ethics committee, France, approved the study protocol (IRB 00008526). Written informed consent was obtained from all the participants or their proxies.

### Study catheters

All DCs were non-tunnelled, non-antimicrobial-impregnated, double-lumen temporary catheters that were only used for RRT or plasma exchange (PE). The site of DC placement, the use of ultrasound guidance for DC insertion, and the decision to replace DCs by VPI or by GWE was at the discretion of operator. The GWE procedure was adapted from Seldinger’s technique (Additional file 1). The procedure for DC insertion and manipulation is described in Additional file 2. At DC removal, DC tips were cultured using a simplified quantitative broth dilution technique with vortexing or sonication. In patients who kept the DC after ICU discharge, paired blood samples were drawn simultaneously from the DC hub and a peripheral vein before discharge to determine the differential time to positivity.

### Definitions

DC-tip colonization, catheter-related bloodstream infection (CRBSI) and DC dysfunction were defined according to French and American guidelines [14, 18].

### Study design

The study included two different cohort analyses. In the first study, we compared DC colonization and dysfunction in patients with or without GWE for DC placement. In patients with multiple DC placements by GWE, only the first DC inserted by GWE was taken into account. The patients were selected by a matched-cohort approach, and matching was performed with replacement. Matching criteria were selected to exclude other factors that could influence catheter infection or dysfunction: severity of illness scoring by simplified acute physiology score (SAPS) II +/-10; insertion site for femoral placement; insertion site and side position for internal jugular placement and duration between ICU admission and DC placement (+/- 2 days for DC inserted <7 days, +/- 5 days for DC inserted from 7 to 15 days, +/- 7 days for DC inserted from 15 to 21 days, +/- 10 days for DC inserted >21 days). As GWE is mostly performed to replace a malfunctioning DC, a second analysis was conducted to compare the rate of DC dysfunctions in new placements by GWE and by VPI in patients from the ELVIS cohort who had consecutive DC placements after the old DC had been removed for dysfunction, regardless of placement technique.

### Statistical analysis

Continuous and categorical variables were expressed as number and percentage or as median and interquartile range, respectively. Comparisons were performed by non-parametric (Mann-Whitney) and chi-square tests as appropriate. In the matched-cohort population, the effect of the strategy of DC insertion (GWE vs. VPI) on colonization and on DC dysfunction was estimated with a marginal Cox model, controlling for differences between groups before DC insertion and at DC insertion, selected by stepwise analysis. The statistical unit was the DC. Statistical analyses were performed with SAS statistical software, version 9.3 (SAS Institute Inc., Cary, NC, USA) and R statistical software, version 2.12.1 (R Foundation for Statistical Computing, Vienna, Austria). A *p* value <0.05 was considered significant.

## Results

### Results of the matched-cohort analysis

Of the 2172 DCs recorded in the ELVIS database and used for the intention-to-treat analysis, 272 were inserted by GWE in 205 patients. Of these, 178 could be matched with controls according to the matching criteria and were therefore used as cases. The 178 controls were identified from 150 DCs in 143 patients in the database (matching with replacement). The characteristics of the patients and DCs are given in Tables 1 and 2. In the GWE group, the reason for prior catheter removal was known in 107 cases, and the reason was dysfunction in 97 cases and suspected infection in 10 cases. In the VPI group, 48 DCs were replacement catheters. The reason for catheter removal was known in only nine cases, and this was dysfunction in eight cases and suspected infection in the other.

**Table 1:** Characteristics of patients in the matched cohort

	GWE	VPI	*P* value
	(n = 178)	(n = 178)	
Age, years, median (IQR)	63 (55–73)	67 (56–78)	0.05
Male, *n* (%)	118 (66.3)	92 (64.3)	
BMI, median (IQR)	27.2 (22.9–31.6)	27.1 (24.2–29.7)	0.61
Immunocompromised, *n* (%)	40 (22.5)	36 (20.2)	0.60
Main reason for ICU admission, *n* (%)			
Septic shock	54 (30.3)	55 (30.9)	0.91
Other shock	21 (11.8)	26 (14.6)	0.43
Coma	13 (7.3)	13 (7.3)	1.00
Acute respiratory failure	38 (21.3)	51 (28.7)	0.11
Acute renal failure	28 (15.7)	16 (9)	0.05
SAPS II, median (IQR)	66 (53–84)	66 (54–81)	0.99
SOFA score, median (IQR)	18 (14-21)	18 (15-20)	0.77
Invasive MV, *n* (%)	47 (26.4)	51 (28.7)	0.64
NIV, *n* (%)	14 (7.9)	11 (6.2)	0.53
DCs/patient, median (IQR)	2.5 (2-3)	1 (1-2)	<0.01
Type of sessions, median (IQR)			
Number of PE sessions	0 (0–0)	0 (0–0)	-
Number of RRT sessions	7.5 (4–14)	4 (2–9)	<0.01
Number of IHD, sessions	3 (1–8)	2 (0–5)	<0.01
Number of CRRT days	2 (0–6)	1 (0–3)	<0.01
Length of stay, median (IQR)			
ICU	19 (11–32)	17 (8–25)	0.04
Hospital	37.5 (21–61)	32.5 (15–54)	0.02
ICU mortality, *n* (%)	68 (38.2)	77 (43.3)	0.33
Hospital mortality, *n* (%)	81 (45.5)	87 (48.9)	0.52

*BMI* body mass index, *GWE* guidewire exchange, *ICU* intensive care unit, *IQR* interquartile range, *CRRT* continuous renal replacement therapy, *IHD* intermittent haemodialysis, *MV* invasive mechanical ventilation, *NIV* non-invasive ventilation, *PE* plasma exchange, *SAPS* simplified acute physiology score, *SOFA* sequential organ failure assessment, *VPI* venipuncture insertion

**Table 2:** Characteristics of dialysis catheters in the matched cohort

	GWE	VPI	*P* value
	(n = 178)	(n = 178)	
Days from ICU admission			
to DC placement, median (IQR)	4 (2–10)	3.5 (1–9)	0.03
MV at DC insertion, *n* (%)	134 (75.3)	133 (74.7)	0.90
Catecholamine at DC insertion, *n* (%)	104 (58.4)	117 (65.7)	0.16
Presence of another catheter			
at the time of insertion, *n* (%)	169 (94.9)	166 (93.3)	0.5
First DC placement, *n* (%)	39 (21.9)	130 (73)	<0.01
Rank of DC placement, median (IQR)	2 (2–2)	1 (1–2)	<0.01
Insertion site, *n* (%)			
Internal jugular	49 (27.5)	49 (27.5)	1.00
Femoral	129 (72.5)	129 (72.5)	1.00
Right side	110 (61.8)	113 (63.8)	0.74
Experience of the operator			
<50 procedures, *n* (%)	135 (75.8)	127 (71.8)	0.18
Alcohol-based skin antiseptic solution, *n* (%)			
5 % povidone-iodine, 70 % ethanol	82 (46.1)	79 (44.4)	0.75
0.5 % chlorhexidine, 67 % ethanol	93 (52.2)	95 (53.4)	0.83
Systemic antimicrobials			
at catheter insertion, *n* (%)	149 (83.7)	151 (84.8)	0.77
DC use, *n* (%)			
No RRT/PE performed	7 (3.9)	8 (4.5)	-
PE only	9 (5.1)	6 (3.4)	-
RRT only	158 (88.8)	164 (92.1)	0.19
Both PE/RRT	4 (2.2)	0 (0)	-
Reason for DC removal, *n* (%)			
DC dysfunction	67 (37.6)	28 (15.7)	<0.01
Suspected DC infection	20 (11.2)	14 (7.9)	-
DC no longer needed	40 (22.5)	58 (32.6)	-
Death of the patient	28 (15.7)	50 (28.1)	-
Other reasons	23 (12.9)	28 (15.7)	-
DC use duration, days, median (IQR)	4 (2–7)	5 (2–8)	<0.01
DC days, *n*		922	937
DC tip culture performed at removal, *n* (%)	154 (86.5)	140 (78.7)	
DC-related colonization, *n* (%)	10 (5.6)	10 (5.6)	1.00

*DC* dialysis catheter, *GWE* guidewire exchange, *ICU* intensive care unit, *IQR* interquartile range, *CRRT* continuous renal replacement therapy, *IHD* intermittent haemodialysis, *MV* invasive mechanical ventilation, *NIV* non-invasive ventilation, *PE* plasma exchange, *RRT* renal replacement therapy, *SAPS* simplified acute physiology score, *SOFA* sequential organ failure assessment, *VPI* venipuncture insertion

The time from admission to DC insertion was shorter in the VPI group than in the GWE group (*p* = 0.03). DCs were mainly inserted on the day of inclusion in the ELVIS study (*p* < 0.01), and DC stay was longer (*p* < 0.01).

### Infection

**Fig. 1:**
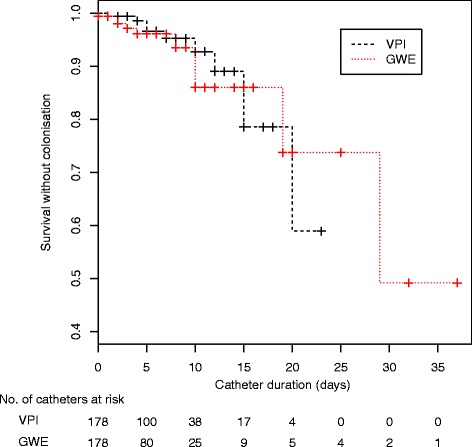
Overall Kaplan-Meier curve of time to dialysis catheter colonization at removal. *GWE* guidewire exchange, *VPI* venipuncture insertion

The DC colonization rate was 5.6 % (10 events) in both groups. After adjustment for baseline differences between groups, ICU stay before insertion and first DC placement were not associated with colonization (weighted hazard ratio (HR) 1.33, 95 % CI 0.57, 3.12; *p* = 0.51 for ICU stay before insertion and weighted HR 4.71, 95 % CI 0.03, 647.07; *p* = 0.51 for the first DC placement). As shown in Fig. 1, GWE was not associated with an increased risk of colonization (10.85 vs. 10.67 per 1000 catheter-days; weighted HR 4.11, 95 % CI 0.14, 122.32; *p* = 0.41). The aetiologic organisms of DC colonization are shown in Table 3. CRBSI was identified in three cases, two in the GWE group and one in the VPI group.

**Table 3:** Distribution of the microorganisms involved in dialysis catheter colonization

Aetiologic microorganisms	GWE	VPI
Colonized DC	N = 10	N = 10
Gram-positive		
*Staphylococcus aureus*	0	1
*Staphylococcus epidermidis*	4	0
Other coagulase-negative *Staphylococci*	2	7
*Enterococcus* species	0	1
Other Gram-positive	1	0
Gram-negative		
*Escherichia coli*	1	0
*Klebsiella pneumonia*	0	1
Fungi	2	1

*DC* dialysis catheter, *GWE* guidewire exchange, *VPI* venipuncture insertion

### Dysfunction

**Fig. 2:**
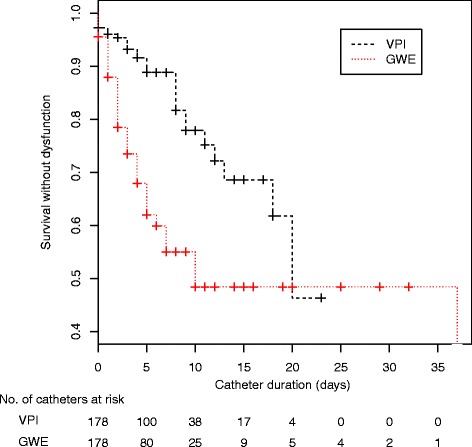
Overall Kaplan-Meier curve of time to dialysis catheter dysfunction at removal. *GWE* guidewire exchange, *VPI* venipuncture insertion

The dysfunction rate was 37.6 % in the GWE group (67 events), and 15.7 % (28 events) in the VPI group (weighted HR 3.67, 95 % CI 2.07, 6.49; *p* < 0.01). After adjustment for baseline differences between groups, ICU stay before insertion and first DC placement were not associated with dysfunction (weighted HR 0.86, 95 % CI 0.70, 1.05; *p* = 0.14 for ICU stay before insertion and weighted HR 0.50, 95 % CI 0.18, 1.34; *p* = 0.16 for the first DC placement). As shown in Fig. 2, GWE led to a significant increase in the risk of dysfunction (72.7 vs. 22.4 per 1000 catheter-days; weighted HR 3.56, 95 % CI 1.66, 7.63; *p* < 0.001).

### Results of the cohort study on sequential DC replacements for malfunctioning DCs

Consecutive dialysis catheterizations with the old DC removed for dysfunction were identified in 301 cases (Additional file 3). The DC dysfunction rate was 49.7 % (80 events, 94.9 per 1000 catheter-days) for new DCs inserted by GWE and 27.9 % (39 events, 36.3 per 1,000 catheter-days) for new DCs inserted by VPI (HR 2.40, 95 % CI 1.66, 3.47; *p* < 0.0001).

After adjustment for side placement and insertion site, dysfunction of new DCs inserted was associated with placement by GWE and placement on the left side but not with insertion site (femoral vs. internal jugular) (Additional file 4).

## Discussion

In this secondary analysis of the ELVIS study, we found that inserting a DC by GWE (as opposed to VPI) did not increase the risk of DC colonization but was associated with a higher risk of DC dysfunction. The risk of DC dysfunction was more than twofold higher when the previous DC was malfunctioning and had been replaced by GWE rather than by VPI.

In a pilot study Palmer et al. demonstrated that guidewire contamination during central line placement predisposes to subsequent colonization of the inserted catheter [19]. This is why replacement by GWE of a non-tunnelled catheter that is suspected to be infected is discouraged, but it may be used to replace a malfunctioning catheter when there is no evidence of catheter infection [14].

Three recent observational studies of critically ill adult patients, designed to assess the impact of catheter replacement by GWE on the risk of infections, yielded conflicting results [20–22]. In a prospective multicentre survey of 1598 central venous catheters, including 67 inserted by GWE, GWE was identified as an independent risk factor of catheter-related bacteraemia; however, more than 20 % of the catheters were inserted outside of the ICU [23]. Conversely, two single-centre observational studies, including one that examined triple-lumen antimicrobial surface-treated catheters [22], reported that the rate of catheter colonization in serial catheter insertions was not influenced by GWE [20, 22]. However, whether the results of these studies can be applied to DC placement by GWE is questionable because central venous catheters and DCs are inserted for different purposes and their extent and manner of use are not the same, and critically ill patients with AKI requiring RRT have a higher mortality rate than the general ICU population.

DC replacement by GWE may have several theoretical advantages over VPI. First, inserting DCs by the blind anatomical landmark technique risks arterial puncture and other mechanical complications [8]. Ultrasound-guided catheterization reduces but does not eradicate these risks, while GWE eliminates puncture-related complications. Second, using a new site may compromise the vascular network. Third, GWE may be the last option when no alternative insertion sites other than subclavian sites are available. There is a paucity of information on the impact of DC placement by GWE on DC colonization in critically ill patients. In a recently published study, no difference in the risk of DC colonization between GWE and VPI was found in 96 patients with initial femoral DC insertion, who underwent serial DC placement by GWE (53 DCs) or VPI (100 DCs) [12].

Our study differed in the severity of illness among the patients, the systematic culture of DC tip at DC removal and the bacteriological method used for defining DC colonization, but it yielded similar results. Our study also provides new data by including both patients with non-femoral dialysis catheterization and patients receiving intermittent haemodialysis.

The choice between GWE and VPI in critically ill patients requiring RRT for DC replacement should also be influenced by the risk of DC dysfunction. Most definitions and studies of DC dysfunction were developed for and conducted in patients with ESRD on intermittent haemodialysis and are difficult to apply to critically ill patients [23]. Data on DC dysfunction in the ICU setting are scarce [9, 10, 17, 24]. Comparison of these studies is difficult because of differences in populations, techniques used for RRT, type of interdialytic DC locks and definitions of dysfunction [25]. The rates of DC dysfunction observed in our study in the VPI group, both in the main analysis (14 %) and in the second analysis that dealt specifically with patients with serial DC replacements (27.1 %), were in agreement with those published in a study using DC removal to define dysfunction (10 % for first DC placements and 24 % for subsequent placements at a new site) [10].

To the best of our knowledge, this study is the first to assess the risk of DC dysfunction when DCs are placed by GWE rather than by VPI in the ICU setting. Several characteristics may predispose to DC dysfunction such as catheter material [26], length [10], gauge [27], insertion site [10] and blood flow velocity through the DC [10]. In the ELVIS study the DC characteristics and causes of DC dysfunctions were not recorded, which restricted our ability to elaborate on the mechanisms involved. However, in each centre only one DC brand and one type of DC material were used. Furthermore, in accordance with local standard practice, almost all internal jugular DCs placed on the right side were 16 cm long and almost all femoral DCs were at least 20 cm long. As controls and cases were matched by centre and insertion site, this suggests that in our study DC characteristics had only a marginal impact on the observed difference in dysfunction rates between VPI and GWE. We speculate that replacing malfunctioning DCs by GWE rather than by VPI more often fails to resolve dysfunction when dysfunction results from incorrect tip location or progressive occlusion of the DC lumen by thrombus.

Our results are in agreement with those of the Cathedia study, which reported that jugular site placement did not outperform femoral site for dysfunction of short-term DCs in the ICU. However, in the Cathedia cohort left-jugular DC insertion conferred a higher risk of dysfunction than other placements [10], whereas in our study left-side insertion in both jugular and femoral positions predisposed to DC dysfunction, probably because it involves a less direct route to the superior vena cava. In our study, DCs were inserted in the femoral site in more than 70 % of cases and mainly on the right side. We cannot exclude that 20-cm-long DCs placed at the left femoral site may not be long enough to allow their tips to extend to the inferior vena cava, contributing to the observed greater risk of dysfunction.

### Limitations

Our work has certain limitations. First, the choice as to whether to perform a GWE or a VPI was at the discretion of the treating physician, and the reasons for the choice were not recorded in the ELVIS database. Thus, no specific adjustment could be performed to take into account these criteria. In our study, in accordance with recommended guidelines [14], GWE was rarely performed for replacement of a DC with suspected infection. Second, as a non-randomized study, it is potentially subject to bias in patient selection. However, the results were probably not affected because of the matched-control approach, which balanced the main risk factors for DC colonization between both groups. Third, we cannot exclude the possibility that we failed to detect an increased risk in DC colonization after GWE because the study did not have sufficient power. However, the sample size in this secondary analysis of the ELVIS study was substantially larger than that of the previously published study, which found similar results. Fourth, including initial DC insertion in the control group may have biased the study against cases. However, several studies suggest the risk of infectious complication is not influenced by initial or subsequent catheterizations but rather by catheter dwell time and longer ICU stay [12, 20]. The putative bias related to initial DC placement had probably only a marginal impact on our results on DC colonization, as controls were adjusted to cases according to ICU stay before DC insertion. In addition, the sensitivity analyses were limited to serial catheterization and yielded similar results. Fifth, the primary endpoint of the study was not CRBSI, which may limit the clinical relevance of our work. However, the use of catheter-tip colonization as a surrogate endpoint for the most severe forms of catheter infection has been widely documented elsewhere [28]. Sixth, our results do not apply to patients with ESRD in dialysis units or to long-term catheterizations.

## Conclusion

In ICU patients requiring DC replacement, GWE does not present a significant risk factor for DC-related colonization/infection but predisposes to dysfunction. It would now be opportune to carry out a randomized controlled study to confirm that GWE could be an acceptable alternative to reinsertion at a different site for preserving the vascular network, particularly in patients with difficult venous access.

### Abbreviations

AKI, acute kidney injury; CRBSI, catheter-related bloodstream infection; DC, dialysis catheter; ESRD, end-stage renal disease; GWE, guidewire exchange; HR, hazard ratio; KDIGO, Kidney Disease Improving Global Outcome; PE, plasma exchange; RRT, renal replacement therapy; SAPS, simplified acute physiology score; SOFA, sequential organ failure assessment; VPI, venipuncture insertion

## Additional files

Additional file 1: Protocol for DC insertion, care and dressing. (DOC 23 kb) Additional file 2: GWE technique for DC placement. (DOC 23 kb) Additional file 3: Follow up of DCs inserted by GWE or by VPI at a new site to replace a previous one removed for dysfunction. (DOCX 26 kb) Additional file 4: Factors associated with DC colonization in the 38 pairs of consecutive DC placements when the first DC was colonized at removal. (DOCX 15 kb)
