# Supplementary material for: Guidewire exchange vs new site placement for temporary dialysis catheter insertion in ICU patients: is there a greater risk of colonization or dysfunction?
Source: Crit Care. 2016 Jul 30;20:230. doi: 10.1186/s13054-016-1402-6 (PMC4967331; doi:10.1186/s13054-016-1402-6)
Supplement: Additional file 1: — Protocol for DC insertion, care and dressing. (DOC 23 kb) [file 13054_2016_1402_MOESM1_ESM.doc]

Supplemental digital content 1. Protocol for DC insertion, care and dressing.

DC insertion and handling were performed in all study centres using maximal sterile barrier precautions according to French recommendations for catheter insertion care and maintenance, which are similar to CDC recommendations. Skin antisepsis at DC insertion and during dressing changes was performed using alcoholic povidone iodine solution or alcoholic chlorhexidine. A semipermeable transparent polyurethane dressing was applied over the insertion site and surrounding skin. Neither antiseptic ointment nor antiseptic-impregnated dressings were used. Dressings were changed 24 hours after DC insertion then every three days. Leaking or soiled dressings prompted immediate dressing change. No DC manipulations were allowed during the inter-RRT/PE periods. DCs were not changed after a fixed insertion time. DCs were removed if no longer needed, if DC-related dysfunction was present, or if a DC-related infection was suspected. When DC-related infection was suspected, one or more peripheral blood samples for culturing were collected within 48 hours before or after DC removal.
