# Supplementary material for: Guidewire exchange vs new site placement for temporary dialysis catheter insertion in ICU patients: is there a greater risk of colonization or dysfunction?
Source: Crit Care. 2016 Jul 30;20:230. doi: 10.1186/s13054-016-1402-6 (PMC4967331; doi:10.1186/s13054-016-1402-6)
Supplement: Additional file 2: — GWE technique for DC placement. (DOC 23 kb) [file 13054_2016_1402_MOESM2_ESM.doc]

Supplemental digital content 2. GWE technique for DC placement

GWE was performed in all study centres using strict aseptic precautions. GWE procedure was adapted from Seldinger’s technique. Briefly, about 5cm of the DC outside the entry site and the DC insertion site were extensively soaked with alcoholic povidone iodine or alcoholic chlorhexidine solution. Sterile drapes were placed around to cover the DC except for the component under manipulation. This component was then covered with alcohol-povidone iodine or alcohol-chlorhexidine impregnated gauze. The DC was clamped above the skin and about 5 cm of the clean subcutaneous part of the DC was withdrawn and then cut using sterile scissors at the distal portion of the clamp. The external cut portion was covered with drapes. The operator then removed his/her gloves and changed to a new pair of sterile ones. The guidewire was gently introduced with the soft J tip first through the distal lumen of the DC so that the entire DC was filled with the guidewire. The clamp was removed with one hand while the other held the external part of the DC. The new DC was then threaded over the guidewire and secured to the skin, as would be the case for a new DC by VPI.
