# Supplementary material for: Guidewire exchange vs new site placement for temporary dialysis catheter insertion in ICU patients: is there a greater risk of colonization or dysfunction?
Source: Crit Care. 2016 Jul 30;20:230. doi: 10.1186/s13054-016-1402-6 (PMC4967331; doi:10.1186/s13054-016-1402-6)
Supplement: Additional file 3: — Follow up of DCs inserted by GWE or by VPI at a new site to replace a previous one removed for dysfunction. (DOCX 26 kb) [file 13054_2016_1402_MOESM3_ESM.docx]

Supplemental digital content 3. Follow-up of DCs inserted by GWE or by VPI at a new site to replace a previous one removed for dysfunction

2185 DCs

161 new DCs placed by GWE

516 DCs removed for dysfunction

301 DCs replaced by a new DC at removal

140 new DCs placed by VPI

80/161 (49.7%) removed for dysfunction

39/140 (47.9%) removed for dysfunction

DC, dialysis catheter; GWE, guidewire exchange; VPI, venipuncture insertion (at a new site)
