# Supplementary material for: Guidewire exchange vs new site placement for temporary dialysis catheter insertion in ICU patients: is there a greater risk of colonization or dysfunction?
Source: Crit Care. 2016 Jul 30;20:230. doi: 10.1186/s13054-016-1402-6 (PMC4967331; doi:10.1186/s13054-016-1402-6)
Supplement: Additional file 4: — Factors associated with DC colonization in the 38 pairs of consecutive DC placements when the first DC was colonized at removal. (DOCX 15 kb) [file 13054_2016_1402_MOESM4_ESM.docx]

Supplemental digital content 4: Factors associated with DC colonization in the 38 pairs of consecutive DC placements when the first DC was colonized at removal

Variables HR (95% CI) p value

GWE 2.4 (0.68-8.66) 0.17

Side of insertion

Right 1 (Reference) -

Left 0.24 (0.05-1.07) 0.06

Site of insertion

Internal jugular 2.03 (0.66-6.18) 0.21

Other 1 (Reference) -

HR, hazard ratio

Dash indicates that no p value can be given since this is the reference variable
